# Cluster randomised-control trial for an Australian child protection education program: Study protocol for the *Learn to be safe with Emmy and friends*™

**DOI:** 10.1186/s12889-016-2721-x

**Published:** 2016-01-25

**Authors:** Codi White, Dianne C. Shanley, Melanie J. Zimmer-Gembeck, Katrina Lines, Kerryann Walsh, Russell Hawkins

**Affiliations:** 1 School of Applied Psychology and Menzies Health Institute of Queensland, Griffith University, Gold Coast, Australia; 2 Act for Kids, Brisbane, Australia; 3 Faculty of Education, Queensland University of Technology, Brisbane, Australia; 4 College of Healthcare Sciences, James Cook University, Cairns, Australia

**Keywords:** Prevention, Child protection education, School-based program, Cluster randomised controlled trial, Protocol

## Abstract

**Background:**

Child maltreatment has severe short-and long-term consequences for children’s health, development, and wellbeing. Despite the provision of child protection education programs in many countries, few have been rigorously evaluated to determine their effectiveness. We describe the design of a multi-site gold standard evaluation of an Australian school-based child protection education program. The intervention has been developed by a not-for-profit agency and comprises 5 1-h sessions delivered to first grade students (aged 5–6 years) in their regular classrooms. It incorporates common attributes of effective programs identified in the literature, and aligns with the Australian education curriculum.

**Methods/Design:**

A three-site cluster randomised controlled trial (RCT) of Learn to be safe with Emmy and friends™ will be conducted with children in approximately 72 first grade classrooms in 24 Queensland primary (elementary) schools from three state regions, over a period of 2 years. Entire schools will be randomised, using a computer generated list of random numbers, to intervention and wait-list control conditions, to prevent contamination effects across students and classes. Data will be collected at baseline (pre-assessment), immediately after the intervention (post-assessment), and at 6-, 12-, and 18-months (follow-up assessments). Outcome assessors will be blinded to group membership. Primary outcomes assessed are children’s knowledge of program concepts; intentions to use program knowledge, skills, and help-seeking strategies; actual use of program material in a simulated situation; and anxiety arising from program participation. Secondary outcomes include a parent discussion monitor, parent observations of their children’s use of program materials, satisfaction with the program, and parental stress. A process evaluation will be conducted concurrently to assess program performance.

**Discussion:**

This RCT addresses shortcomings in previous studies and methodologically extends research in this area by randomising at school-level to prevent cross-learning between conditions; providing longer-term outcome assessment than any previous study; examining the degree to which parents/guardians discuss intervention content with children at home; assessing potential moderating/mediating effects of family and child demographic variables; testing an in-vivo measure to assess children’s ability to discriminate safe/unsafe situations and disclose to trusted adults; and testing enhancements to existing measures to establish greater internal consistency.

**Trial registration:**

Australian and New Zealand Clinical Trials Register (ACTRN12615000917538). Registered (02/09/2015).

## Background

Child maltreatment is a serious and widespread problem affecting children around the world [1, 2]. The term child maltreatment refers to one or more of 4 major subtypes: physical abuse, emotional (or psychological) abuse, sexual abuse, and neglect. Child maltreatment can involve acts of omission or commission by parents, caregivers, or community members (e.g. teachers, clergy) and may result in death, serious injury, or exploitation [3, 4]. Epidemiological monitoring reveals it is associated with a variety of specific negative outcomes for children, including higher levels of mental illness, poverty, greater engagement with crime, and physical, emotional, mental and social maladjustment across the entire developmental trajectory [5–8].

One way to help children to learn about and safeguard themselves from maltreatment is through school-based child protection education programs, which were first implemented in the USA in the 1980s [9, 10]. Also known as child abuse prevention, child assault prevention, child safety, personal safety, and protective behaviours programs [9–13], these typically focus on teaching severalcore concepts, including how to recognise safe and unsafe situations, how to react when approached, and how to identify safe adults to speak with about unsafe situations [14]. A major benefit of child protection education programs is that, unlike programs focusing solely on one type of harm (i.e. bullying, physical violence, sexual harassment), they equip children with knowledge and skills that may be generalised to recognise, react to and report multiple types of harm and risk situations.

Despite the wide uptake of child protection education programs in countries as diverse as Germany, Hong Kong, Taiwan, Turkey, and the United States [15], systematic reviews and meta-analyses highlight that very few highly rigorous program evaluations exist [15–19]. Indeed, it has been identified that few evaluations have used control groups and randomised designs, whilst fewer still track outcomes for more than 6 months to examine the long-term effectiveness of these programs. There have also been concerns regarding the measures utilised to evaluate outcomes. For example, recommendations for future research designs include the need to measure: a) primary or targeted program outcomes such as self-protective skills, behaviour change, abuse disclosures;b)multiple program outcomes including knowledge, intentions, skills,and program satisfaction,within the one sample; and c) potential negative impacts such as increased fear or anxiety.

This study extends an earlier successful Australian study of a specific child protection education program, *Learn to Be Safe with Emmy*™ [20]. This was a quasi-experimental study in which classrooms were randomised to conditions (program and waitlist); with the waitlist comparison group receiving the program after they completed post-test measures (5 weeks after administration of baseline measures). In this study, a total of 245 first grade students (aged 5–6 years) participated in the *Learn to Be Safe with Emmy*™ program, recruited from five primary (elementary) schools on the Gold Coast, Queensland. The study demonstrated the program was effective in increasing participants’ self-protective knowledge and skills, and their intentions to respond in a safe manner to unsafe situations including hypothetical situations with risks of bullying, abuse and sexual assault, without increasing or decreasing child anxiety.

The current study will extend on Dale et al. [20] in six ways. It will: (i) use a larger sample, with schools from a broader range of demographic areas; (ii) randomise at school-level to prevent cross-learning between conditions; (iii) provide a 6-month follow-up for the waitlist control condition, and 12- and 18-month follow-ups for the intervention condition; (iv) examine the degree to which parents/guardians discuss intervention content with children at home; (v) develop and utilise an in-vivo measure to assess children’s ability to discriminate safe/unsafe situations and disclose their experience to trusted adults; and (vi) enhance the intentions to respond measure by asking about both safe and unsafe situations to test if safety responses (i.e. telling an adult) were used appropriately according to the level of perceived risk.

### Aims

The aim of this study is to conduct an empirical assessment, using a cluster randomised-controlled trial [21], of key outcomes of the *Learn to be safe with Emmy and friends*™ program. Specifically, the current study will test the following five hypotheses:

1. When compared to children in the waitlist condition for the same period, children in the intervention condition will show the following changes from pre-test to post-test:
  - significantly greater increase in knowledge of interpersonal safety
  - significantly greater ability to distinguish between safe and unsafe situations
  - significantly greater intention to engage in safe responses to unsafe situations
  - significantly greater confidence to take appropriate action in unsafe situations
2. When compared to parents/guardians of children in the waitlist condition for the same period, parents/guardians of children in the intervention condition will report significantly greater increases in their child’s knowledge of interpersonal safety and use of self-protective skills.
3. Post-intervention, children in the intervention condition will:
  - refuse a simulated in vivo lure by an unfamiliar adult at post-intervention at a higher rate than children in the waitlist condition.
  - disclose the presence of an unfamiliar adult i) earlier; and ii) at a higher rate, than children in the waitlist condition.
4. Children’s post-intervention knowledge of interpersonal safety will be positively correlated with both behavioural intentions, application of self-protective skills, and disclosure.
5. Children’s anxiety scores will not change from pre-intervention to post-intervention.

## Methods/Design

The Human Research Ethics Committees at Griffith University (GU Ref No: PSY/48/14/HREC) approved this study, as did the Research Committee at the Department of Education and Training, Queensland. It is also registered with the Australian and New Zealand Clinical Trials Register (ACTRN12615000917538) and has received Australian Research Council funding under the Linkage Projects scheme (ID: LP130100304) in a partnership between Griffith University, James Cook University, Queensland University of Technology and the not-for-profit organisation, Act for Kids.

The study consists of a three-site cluster randomised control trial using a waitlist-control group. The unit of randomisation will be the school. The unit of analysis will be individual children; however the nesting effects of classes, schools, and site location will be taken into account through hierarchical modelling. Data from parents/guardians about immediate child outcomes will be paired with the respective child data. The main outcome evaluation will be supplemented by a process evaluation using fidelity data from facilitators, session auditors, and satisfaction surveys completed by parents/guardians and teachers.

### Recruitment and participants

The study will be conducted in first grade classrooms with children aged 5–6 years in three education regions in Queensland, Australia: Brisbane (Metropolitan Region, urban schools), Gold Coast (South East Region, urban schools), and Townsville (North Queensland Region, rural schools). Eightschools in each region will be randomly selected to participate. Trained research assistants not involved in data collection will initially contact schools. An information sheet detailing the *Learn to be safe with Emmy and friends*™ program and explaining the evaluation will be sent to school principals who will be asked to provide consent for their school’s participation. Randomisation will be conducted following principal consent, after which time, schools will be asked to distribute standardised information sheets with consent forms (either waitlist-control or intervention version) to parents/guardians (hereafter, parents) of all children enrolled in first grade. Parents return consent forms to the school if they agree for their child to participate.

A simple random sampling strategy with individuals as the primary sampling unit was assumed, and G*Power was then used to estimate sample size. This returned a suggested total sample size of at least 100 children per group at each of three sites. Based on similar studies [22] that have used cluster sampling with schools as the primary sampling unit, the sample size with this design effect was estimated to be approximately 1.5, taking into account potential attrition of (20 %) owing to student absences and/or mobility. In addition, enough power will be needed to examine the effects for subgroups (sex/school/family/stress, etc.). Therefore, a total sample size of at least 1350 is required in this study to test the effectiveness of the intervention. Assuming conservative class sizes of 20 students per class (60 children per school; 9 schools in each region), a final initial sample size of 1620 Year 1 children and their parents is planned to give sufficient power for this study.

### Inclusion/exclusion criteria

Children will be eligible to participate in the study if they are enrolled in a first grade classroom at a participating school. Schools who have taught an alternative child protection education program to their first grade students will be ineligible to participant in this RCT.

### Randomisation

After school recruitment and principal consent, a concealed randomisation process will be undertaken using a computer-generated list of random numbers. Entire schools will be randomised to intervention or control to prevent knowledge and skill sharing among students across conditions. Researchers conducting data collection and data analysis will be blinded to group membership.

### Intervention: Learn to *b*e *s*afe with Emmy and friends™

*Learn to be safe with Emmy and friends*™ is a child protection education program designed for first grade students [23]. It consists of five 1-h sessions provided free-of-charge by trained facilitators, delivered in children’sregular classrooms once per week for five weeks. A unique program feature is the presence of an adult-size program mascot called “Emmy” who, as a child-like character, assists program facilitators by drawing attention to key themes, and modelling strategies in developmentally-appropriate ways. The *Learn to be safe with Emmy and friends*™ program addresses many of the core themes common toeffective child protection and bullying prevention programs including 1) teaching children how to recognise potentially dangerous people or situations, 2) teaching children how to respond (i.e. with rehearsed statements and positive actions) and 3) encouraging children to report or disclose past, current or future abuse or unsafe encounters to trusted authority figures [24–27]. The main topics for each of the five *Learn to be safe with Emmy and friends*™ sessions are shown in Table 1.

**Table 1:** Overview of the *Learn to be safe with Emmy and friends*™ Program Content and Purpose

Session	Content and Purpose
1	Emotion Recognition and Early Warning Signs. Content focuses on body signals that indicate various emotions as well as emotions or feelings that might indicate danger. Strategies to assist in remaining calm and reducing anxiety or worry are also taught. This is so that children are able to recognise when they might be feeling scared or angry, and know how to calm down so that they can more effectively respond to situations.
2	Early Warning Signs and Safe/Unsafe Situations. Content focuses on early warning signs (i.e., indicators given by the body that a situation may be unsafe), discriminating safe and unsafe situations, and identifying situations that may feel unsafe but are either fun (e.g. roller coasters) or within the child’s control (e.g. a test).
3	Personal Space and Private Body Areas. Content addresses the concept of personal space and private body areas as well as teaching responses to personal space violations and information regarding disclosure.
4	Safe/Unsafe Secrets. Content focuses on examples of safe and unsafe secrets and distinguishing between these. Encouragement is also given to disclosing potentially unsafe secrets to safe adults.
5	Identification of Safe Adults and Safety Networks for Disclosure. The final session focuses on recognising and developing networks of safe adults from whom children may seek help. This includes personal networks individual to each child as well as provision of contact information for specialist services for children such as Kids Help Line.

*Learn to be safe with Emmy and friends*™ incorporates many of the delivery methods identified in effective psycho-education programsincluding active participation (i.e. role play), explicit training (specification and rehearsal of desired behaviours), standardised materials, and repeated presentations and summaries of materials as well as program materials sent home for parental review [16, 26, 28]. The program also aligns with the *Australian Curriculum: Health and Physical Education* [29].

### Control group

Waitlist-control schools will receive the school curriculum as usual and will be waitlisted for the intervention. They will participate in the pre-assessment (baseline), post-assessment (5 weeksafter baseline) and six-month follow-up data collection points for the evaluation. They will commence the program after the six-month follow-up assessment has been completed.

### Child measures

Trained research assistants will administer all child measures during individual verbal interviews at participating schools. Measures to be administered will include children’s knowledge of program concepts, intentions to use program knowledge, skills, and help-seeking strategies, actual use of program material in a simulated situation, and anxiety as a result of program participation.

#### Protective behaviours questionnaire [20]

The ProBeQ is a self-report 12-item questionnaire assessing children’s knowledge of the six core interpersonal safety concepts covered in the five *Learn to be safe with Emmy and friends*™ program lessons: (i) emotion recognition, (ii) early warning signs, (iii) private and public body parts, (iv) personal space, (v) safe and unsafe secrets and (vi) identification of safe adults as well as the program mantra ‘tell, tell, tell again until someone listens and helps’. Scores will be summed to provide a Total Protective Behaviours Knowledge score (maximum score of 22) with higher scores indicating greater knowledge of the concepts [20]. Because the ProBeQ is a test of knowledge with potential answers that range in their content and structure, psychometric properties are not calculated.

#### Application of Protective Behaviours Test-Revised (APBT-R)

The APBT-R will be administered both at pre- and post-test evaluation. This is a revised version of the original APBT developed by Dale et al. [20] as a six-item, child-report measure assessing children’s intention to apply self-protective knowledge and skills, and seek appropriate help in unsafe situations. In the APBT-R, unsafe scenarios were retained and two new safe scenarios were added. Two developmental psychologists and two child abuse prevention experts screened new safe scenarios. The APBT-R thus includes two safe and four unsafe scenarios presented to children by interviewers as verbal narratives accompanied by simple line drawings. Children’s responses for unsafe scenarios will be rated according to the degree to which they are self-protective. Scores for each scenario will be summed across unsafe scenarios to provide a Total Self-Protective Intention score with higher scores indicating greater protective behaviour intentions. Children will also be asked to rate for all scenarios whether or not each scenario is safe on a yes/no response. These responses will be marked as correct or incorrect and summed to further provide a Total Risk Accuracy score with higher scores indicating greater accuracy in classifying scenarios.

#### Observed Protective Behaviours Test (OPBT)

This will be administered to children in the control and intervention conditions at either post-test evaluation or the six-month evaluation. A structured two-part in vivo behavioural situation has been developed by the first author expressly for this study and will be used to test children’s ability to implement the safety skills learned during *Learn to be safe with Emmy and friends*™. Part 1 will measure recognition of unsafe situations and theapplication of relevant safety skills. Children will be presented with a simulated unsafe situation enacted by an unfamiliar confederate. In the script for the unsafe situation after greeting the child, the interviewer will leave the room under the pretext of making a telephone call [30]. During the interviewer’s absence,a confederate will enter the room and ask the child to leave with them. The confederate will then remember that they have forgotten a bag and leave, asking the child to keep their presence a secret and dropping a pen to the floor as they leave. The manipulation will take approximately 1 min with the interviewer returning within 30 s of the confederate exiting and resuming the interview.

Part 2 will test children’s disclosure of the unsafe situation. In this, children will be presented with 6 prompts to disclose the presence of the confederate (e.g., “You probably didn’t notice if the pen was in the room before, did you?”). At the end of the interview, prior to administering the anxiety measure the confederate will return and the secret will be revealed. This will provide an opportunity for the interviewer to model disclosure of the unsafe situation with the child and engage in debriefing. The OPBTis based on behavioural skills training and measures used by Johnson et al. [31, 32] and Gunby et al. [33] in evaluations of children’s personal safety skills, as well as the secret-keeping scenario and seven point secret-keeping scale created by Dunkerley and Dalenberg [30].

#### Revised Children’s Manifest Anxiety Scale 2^nd^ Edition (RCMAS-2 Short Form), [34]

The RCMAS-2 Short Form consisting of 10 yes/no items will be used to examine whether children experience increased or decreased anxiety as a result of program participation. A Total Anxiety score can be calculated and three anxiety sub-scales include, physiological anxiety, worry, and social anxiety. (α = .82), [34]. Test-retest reliability has been calculated at .54 for the total score with a US sample [34] and .61 to .68 with an Asian sample [35].

### Parent measures

All parent measures will be administered as self-report. Measures to be administered will include family demographics, discussion monitor, program concepts checklist, satisfaction questionnaire, and a parent stress inventory.

#### Demographics

Family and child demographic information will be collected in 10 items including child gender, child age, parental gender, parental age, family structure, parental income and parental education. In these items family community engagement and the child’s social and school engagement will also be assessed.

#### Parent discussion monitor

Parents will complete a 4-item measure each week documenting:time spent reviewing the program information sheets with their children at home, time spent discussing information from *Learn to Be Safe with Emmy and Friends*™ lessons with their children and whether the content of the discussion was raised by the child or parent.

#### Parent Protective Behaviours Checklist (PPBC), [20]

The PPBC is a ten-item parent-report measure. For each item, parents’ assesses the extent to which they observed that (i) their children using self-protective skills; and/or (ii) their child understood the six concepts taught in the *Learn to be safe with Emmy and friends*™ program. Parents will indicate using a response scale of 1 (Not at all) to 4 (Always) for self-protective skills, and 1 (Unsure) to 4 (Very sure) for understanding. Scores will be summed to provide a Total PPBC score. This measure has good internal consistency (α = 0.84), [20].

#### Parent Satisfaction Questionnaire (PSQ), [20]

The 3-item PSQ will be used to assess parental subjective satisfaction with the program, perceptions of changes in their child’s presentation or behaviour since program commencement, and any additional comments via open-ended text response.

#### Parent Stress Inventory: Short Form (PSI/SF), [36]

The PSI/SF is a 36-item parent-report measure that assesses stress in parents of children aged 1 month to 12 years. A Total Stress score is calculated from three subscales including Parental Distress, Parent–child Dysfunctional Interaction, and Difficult Child. The PSI has acceptable test-retest reliability (*r* = .84) and good validity and internal consistency (α = .91), [36].

### Program performance measures

Measures of attendance and program compliance will be used in the program’s process evaluation. These will be completed by program facilitators for each of the program sessions and also by trained research assistants during audits of 10 % of all sessions at each site.

#### Attendance list

To evaluate effects due to partial versus complete attendance, program facilitators will complete an attendance record will be taken for every session.

#### End of session checklist

A session checklist [20] will be used to identify the extent to which specific program content and methods were covered as intended in each session.

### Data collection

#### Assessment procedures T1-T5

Data will be collected at three sites (Brisbane, Gold Coast, and Townsville) and five main time points (T1 to T5). The Time 1 (T1) assessment (pre-test) will be conducted one week prior to program delivery. Time 2 (T2) assessment (post-test) will occur one week after program completion for the intervention group and 5 weeks after Time 1 for the waitlist control group. Time 3 (T3), Time 4 (T4) and Time 5 (T5) assessments will be conducted 6 months, 12 months and 18 months after program completion respectively.

#### Program and process evaluation

Two trained facilitators employed by the not-for-profit agency, Act for Kids, will deliver all program sessions. Facilitators have qualifications in early childhood education, psychology, or child protection. First grade teachers will be present during the sessions. At the start of each session an attendance record will be taken and a brief review of the previous session content covered. At the end of each session facilitators will record the concepts covered and any factors impacting that session’s program delivery. In 10 % of sessions a research assistant will attend to audit the session content.

### Planned analyses

Mixed factorial analyses of variance (ANOVAs) will be used to examine the effectiveness of the program for primary outcomes. Analyses will be conducted to assess differences between the intervention and waitlist control conditions over time using 2 (Group: intervention, waitlist-control) x 3 (Time: T1, T2, T3) mixed ANOVAs. Primary or target outcomes include children’s knowledge of program concepts, intentions to use program knowledge, skills, and help-seeking strategies, and anxiety as a result of program participation. T-tests will be conducted to examine the difference between groups in use of program skills and disclosure during the simulated situation conducted at T2 and T3.

Following the 6-month follow-up of the children who received the program, data will be analysed to assess stability of improvement in the treatment group from post to, 6-, 12- and 18-month follow-up. One-way ANOVAs will also be conducted for these variables for the intervention group across the five time points (T1, T2, T3 and T4, T5).

Hierarchical linear modelling [37] will be used to examine any effects of the hierarchical structure of the study design. If there are hierarchical effects, this analysis will be used to account for influences of classroom (level 2) and school level effects (level 3), while examining the effects of condition (Emmy/waitlist) and within student changes over time. Regression will be used to test the mediation analyses and to examine any effects of moderators (e.g. demographic variables, parental stress, dose–response) to determine which factors may differentiate intervention effects. Intent-to-treat analyses will also be conducted to account for potential effects of differential drop-out.

## Discussion

This RCT will assess the effectiveness of the *Learn to be safe with Emmy and friends*™ program administered to first grade children in three regions in the state of Queensland, Australia. This program will be rigorously evaluated in the first multi-site cluster randomised controlled trial of a school-based child protection education program conducted in Australia. This child protection education program targets first grade children (aged 5–6 years), and draws on a set of core concepts identified in child protection and bullying prevention programs. Several key program outcomes will be assessed at multiple stages. We hypothesise that children’s short-term (T1 to T2) knowledge of program concepts,intentions to use program knowledge, skills, and help-seeking strategies, and actual use of program material in a simulated situation will increase. We also hypothesise that children will show long-term (T2 to T5) retention of these knowledge and skills. Children’swill also be measured to rule out any potential adverse effects of raising awareness of dangers and risk related to child protection issues. Other key program elements that will be assessed include parental discussion of material, parents’ observations of their children’s use of program materials, parents’ satisfaction with the program, and parental stress. The effectiveness of this program will also be evaluated for moderating effects, which will determine whether adaptations are required for different populations. Few experimental evaluations of a child protection education program have ever been reported in Australia (see [20], for an exception); this study, therefore, will fill an important gap in the child maltreatment prevention literature and will provide robust evidence of the benefits or otherwise of school-based child protection education programs in this context. The *Learn to be safe with Emmy and friends*™ program, while delivered selectively to volunteering schools at present, may have potential to become a more widely-used program should its effectiveness be determined.
